# *WBSCR22* confers oxaliplatin resistance in human colorectal cancer

**DOI:** 10.1038/s41598-017-15749-z

**Published:** 2017-11-13

**Authors:** Dongmei Yan, Linglan Tu, Haining Yuan, Jianfei Fang, Liyan Cheng, Xiaoliang Zheng, Xiaoju Wang

**Affiliations:** The Center for Molecular Medicine, Zhejiang Academy of Medical Sciences, Hangzhou, 310013 Zhejiang, China

## Abstract

Human *WBSCR22* gene is involved in tumor metastasis, cell growth and invasion, however, its role in chemosensitivity to antitumor agents remains unknown. In this study, we analyzed the TCGA cohort and found the expression of *WBSCR22* was significantly elevated in human colorectal cancer (CRC) tissue. *WBSCR22* could be served as an independent risk predictor for overall survival (OS), and up-regulated *WBSCR22* could predict unfavorable OS for CRC patients. Knockdown of *WBSCR22* significantly sensitized CRC cells to oxaliplatin *in vitro* and *in vivo*, while overexpression of *WBSCR22* led to cellular resistance to oxaliplatin treatment. Although *WBSCR22* knockdown did not change cell cycle, it increased the oxaliplatin-induced cellular apoptosis. *WBSCR22* knockdown augmented the oxaliplatin-induced intracellular reactive oxygen species (ROS) production and ROS-induced 8-oxoguanine (8-oxoG) oxidative lesion accumulation, likely sensitizing oxaliplatin treatment. These results demonstrate that *WBSCR22* is involved in CRC resistance to oxaliplatin, suggesting *WBSCR22* may represent a novel oxaliplatin resistance biomarker as well as a potentail target for CRC therapeutics.

## Introduction

Human *WBSCR22* gene was initially identified as one of 26 genes deleted in Williams-Beuren syndrome characterized by dysmorphic facial features, congenital heart and vascular disease, unique cognitive^1–3^. While the mRNA was detected ubiquitously in all tissues, and the protein was markedly expressed in heart, skeletal muscle and kidney^4,5^. WBSCR22 contains a nuclear localization signal and a common S-adenosyl-L-methionine binding motif that is evolutionarily conserved in methyltransferases^6^. However, the specific cellular function of *WBSCR22* is still poorly understood.

*WBSCR22* was over-expressed in invasive breast cancer, and its ectopic expression in non-metastatic cells significantly promoted the metastasis formation by suppressing *Zac1/p53*-dependent apoptosis, but no effects on cell growth and motility^7^. In multiple myeloma, *WBSCR22* was required for the tumor cells to survive^8^, and its product was up-regulated in both primary plasma cells and primary multiple myeloma tumor cells, indicating its roles in plasma cell biology^8^. Knockdown of *WBSCR22* attenuated cell growth and invasive abilities in multiple cells^9,10^. However, *WBSCR22* was significantly down-regulated in lung inflammatory and neoplastic pathologies^11^, suggesting its diverse functions in the context of cellular environment. WBSCR22 was also identified as a novel glucocorticoid receptor (GR) co-modulator by regulating GR recruitment to the genome as well as mediating subsequent histone modification through binding to the histone-associated proteins and protein kinases^11^.

Human colorectal cancer (CRC) is continuously the third most common cancer and the third most common cause of cancer-related death worldwide^12^. Oxaliplatin, a third-generation platinum-based antitumor agent, is widely used as the standard first-line chemotherapy for CRC. However, the development of chemoresistance limits its effectiveness in clinical practice. While many mechanisms were identified for oxaliplatin resistance^13–16^, recent studies showed that DNA hypermethylation, histone post-translational modifications and microRNAs were also involved in chemoresistance^15,17,18^.

In the present study, we analyzed the TCGA cohort and found *WBSCR22* was significantly expressed in human CRC tissue. We further investigated the effects of *WBSCR22* on oxaliplatin sensitivity in CRC, showed that *WBSCR22* knockdown significantly sensitized CRC cell to oxaliplatin *in vitro* and *in vivo*. *WBSCR22* knockdown also induced the cell apoptosis and increased oxaliplatin-induced intracellular ROS production and ROS-induced 8-oxoG oxidative lesion accumulation. The results show that *WBSCR22* induces the chemosensitivity to oxaliplatin in CRC, suggesting it may represent a novel resistance biomarker as well as a potential target for colorectal cancer therapy.

## Results

### *WBSCR22* mRNA was elevated in human CRC and served as an independent prognostic factor for CRC patients

**Figure 1:**
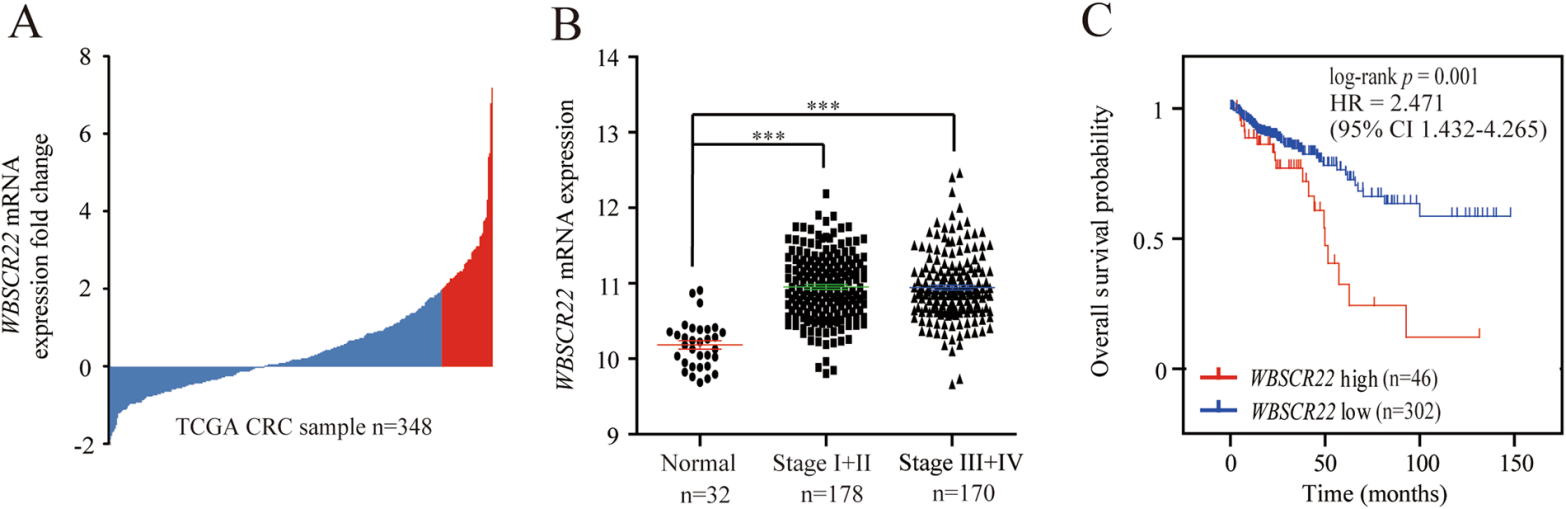
*WBSCR22* gene was over-expressed and predicted a poor clinical outcome in human CRC of the TCGA cohort. (**A**) The expression of *WBSCR22* in 348 clinical CRC specimens in the TCGA cohort. The red bars represented CRC samples having a more than 2-fold higher *WBSCR22* expression than normal samples. (**B**) *WBSCR22* levels were compared between normal samples and different NMT stages of CRC samples. A single spot represented the *WBSCR22* expression value of an individual sample, and the results were expressed as the mean ± SE. ***p < 0.001. (**C**) Kaplan-Meier survival curves were plotted according to the different *WBSCR22* mRNA level of all CRC patients. p values were obtained from log-rank test, while hazard ratio (HR) and 95% confidence interval (CI) were determined by univariate Cox regression model.

We analyzed the mRNA expression profile of *WBSCR22* in an independent TCGA cohort, and found *WBSCR22* had more than 2-fold higher expression in 13% (46/348) of CRC tissues than 32 cases of normal controls (Fig. 1A). It was significantly elevated in CRC tissues, however, there was no significant differences across the TNM stages (Fig. 1B). The association between CRC lymphatic invasion and high *WBSCR22* expression was statistically significant (p = 0.011) (Table 1). Kaplan-Meier analyses showed that all CRC patients with high *WBSCR22* expression had a significantly shorter overall survival(OS) than low expression, therefore, *WBSCR22* could predict significantly unfavorable OS for patients with high *WBSCR22* exporession level (Fig. 1C). Subsequent univariate and multivariate Cox regression analyses were performed to determine the independence of the prognostic value of *WBSCR22*. After correction for clinical characteristics, high *WBSCR22* expression was found to be an independent risk predictor of OS (p = 0.005, HR = 2.391, 95% CI = 1.310–4.365) for CRC patients (Table 2).

**Table 1:** The clinicopathological characteristics of the CRC patients in the TCGA cohort used in this study.

Characteristics	Cases (n = 348)	*WBSCR22* mRNA expression		p
	No. (%)	Low (n = 302)	High (n = 46)	
Gender				
Male	191(54.9)	164(54.3)	27(58.7)	0.577
Female	157(45.1)	138(45.7)	19(41.3)	
Age				
≥60	227(65.2)	196(64.9)	31(67.4)	0.741
<60	121(34.8)	106(35.1)	15(32.6)	
Primary site				
Colon	266(76.4)	234(77.5)	32(69.6)	0.238
Rectum	82(23.6)	68(22.5)	14(30.4)	
Histological type				
Adenocarcinoma	307(88.2)	266(88.1)	41(89.1)	0.837
Mucinous adenocarcinoma	41(11.8)	36(11.9)	5(10.9)	
NMT stage				
Stage I + II	187(53.7)	164(54.3)	23(50.0)	0.585
Stage III + IV	161(46.3)	138(45.7)	23(50.0)	
Venous invasion				
Negative	232(66.7)	207(68.5)	25(54.3)	0.071
Positive	68(19.5)	58(19.2)	10(21.7)	
Unkonw	48(13.8)	37(12.3)	11(24.0)	
Lymphatic invasion				
Negative	213(61.2)	192(63.6)	21(45.7)	0.011
Positive	94(27.0)	80(26.5)	14(30.4)	
Unkonw	41(11.8)	30(9.9)	11(23.9)

**Table 2:** Univariate and multivariate Cox proportional hazards analysis of OS for the CRC patients in the TCGA cohort (HR: hazard ratio, CI: confidence interval).

Characteristics	Univariate analysis		Multivariate analysis	
	p	HR (95% CI)	p	HR (95% CI)
Gender				
Male	0.197	1.000		
Female		0.720(0.437–1.186)		
Age				
≥60	0.018	1.000	0.003	1.000
<60		0.479(0.261–0.879)		0.376(0.199–0.713)
Primary site				
Colon	0.157	1.000		
Rectum		0.599(0.295–1.217)		
Histological type				
Adenocarcinoma	0.001	1.000	0.036	1.000
Mucinous adenocarcinoma		0.405(0.234–0.699)		2.075(1.050–4.101)
TNM stage				
Stage I + II	0.00035	1.000	0.002	1.000
Stage III + IV		2.518(1.517–4.179)		2.419(1.376–4.252)
Venous invasion				
Negative	0.002	1.000	0.235	1.000
Positive		2.604(1.498–4.527)		1.380(0.607–3.138)
Unkonw		1.823(0.939–3.542)		0.401(0.089–1.805)
Lymphatic invasion				
Negative	0.006	1.000	0.471	1.000
Positive		2.314(1.350–3.965)		1.373(0.594–3.173)
Unkonw		1.970(0.965–4.020)		2.679(0.516–13.895)
*WBSCR22*				
Low	0.001	1.000	0.005	1.000
High		2.471(1.432–4.265)		2.391(1.310–4.365)

### The effects of *WBSCR22* knockdown on cell proliferation of CRC cells

To evaluate the significance of *WBSCR22* in CRC, we examined the endogenous WBSCR22 expression in human CRC cell lines by Western blot. The results showed that most CRC cells expressed high WBSCR22 protein, consistent with the TCGA data. We also measured the *WBSCR22* expression in CRC cells with different oxaliplatin sensitivity. Interestingly, the *WBSCR22* expression level was correlated with their oxaliplatin sensitivity (Supplementary Fig. S1).

**Figure 2:**
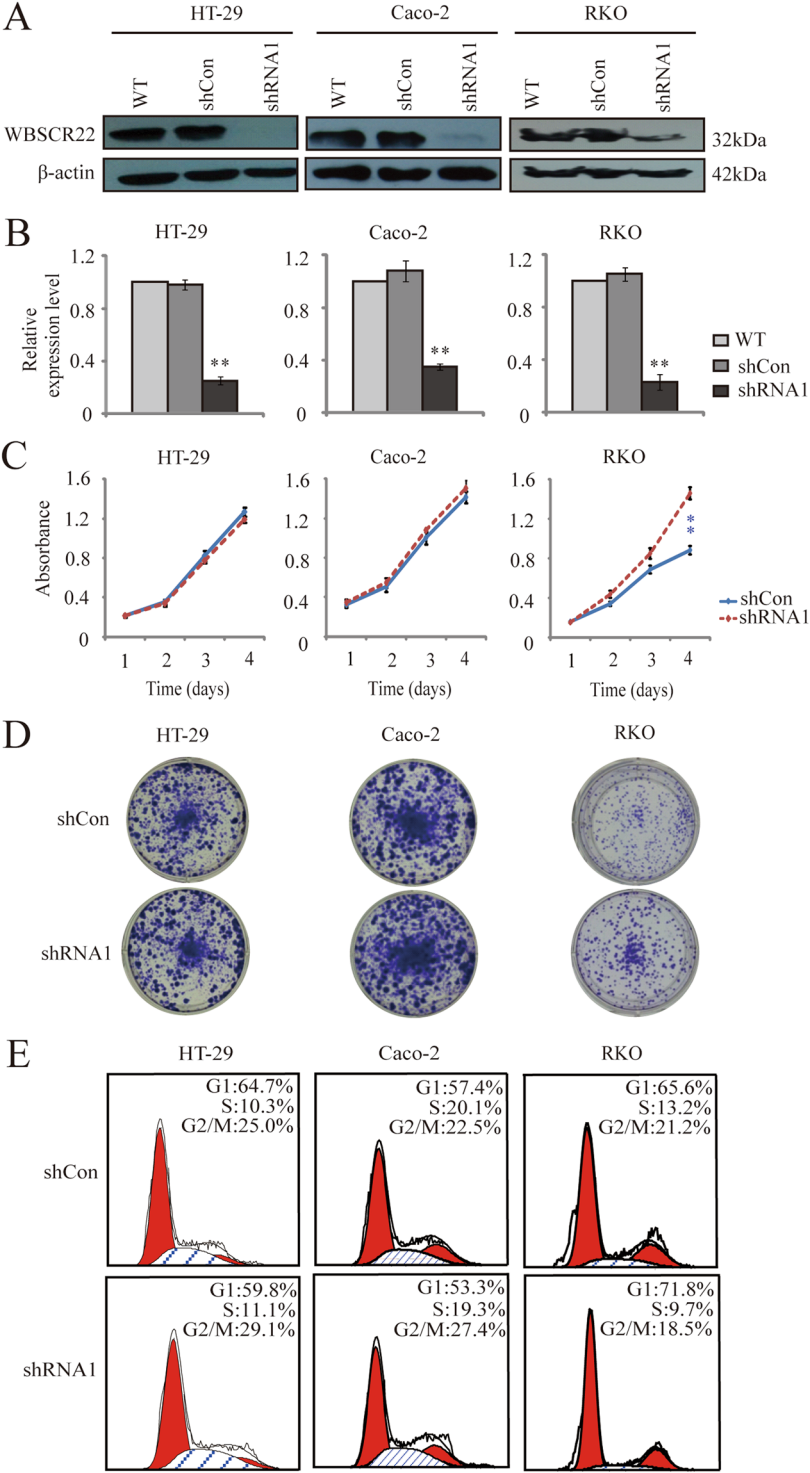
The effects of *WBSCR22* knockdown on cell proliferation and cell cycle of human CRC cells. (**A**) Western blot of WBSCR22 in wild-type (WT), control (shCon), and WBSCR22-knockdown (shRNA1) CRC cells. The β-actin was used as an internal control. (**B**) RT-qPCR analysis of *WBSCR22* expression in WT, shCon, and shRNA1 treated CRC cells. **p < 0.01 (shRNA1 v.s. either WT or shCon). The results were from three independent experiments in triplicate and the data were expressed as the mean ± SD. (**C**) Cell proliferation analysis of the shCon and shRNA1 treated cells by MTT assays. **p < 0.01 (shRNA1 v.s. shCon). The results were representative of three independent experiments in quadruplicate and the results were expressed as the mean ± SD. (**D**) Colony formation assay of the shCon and shRNA1 treated cells. (**E**) Cell cycle distribution by flow cytometry analysis. Percentages of cell populations in each phase of the cell cycle were indicated.

We next knocked down *WBSCR22* gene in HT-29, Caco-2 and RKO cell lines. Western blot and RT-qPCR showed shRNA1 significantly decreased the expression of WBSCR22 at both mRNA and protein levels (60–70% decrease) (Fig. 2A,B). We then assessed the effects of *WBSCR22* knockdown on cell proliferation. For RKO cells, *WBSCR22* knockdown significantly increased the cell proliferation (p < 0.01), and the result was further confirmed by colony formation assay (Fig. 2C,D). For HT-29 and Caco-2 cells, however, there were no significant difference in the cell prolifreation dected by either MTT assay (Fig. 2C) or colony formation (Fig. 2D). Cell flow cytometry, however, showed *WBSCR22* knockdown did not change the cell cycle distributions in all three cell lines (Fig. 2E).

### The effects of *WBSCR22* on oxaliplatin sensitivity *in vitro*

**Figure 3:**
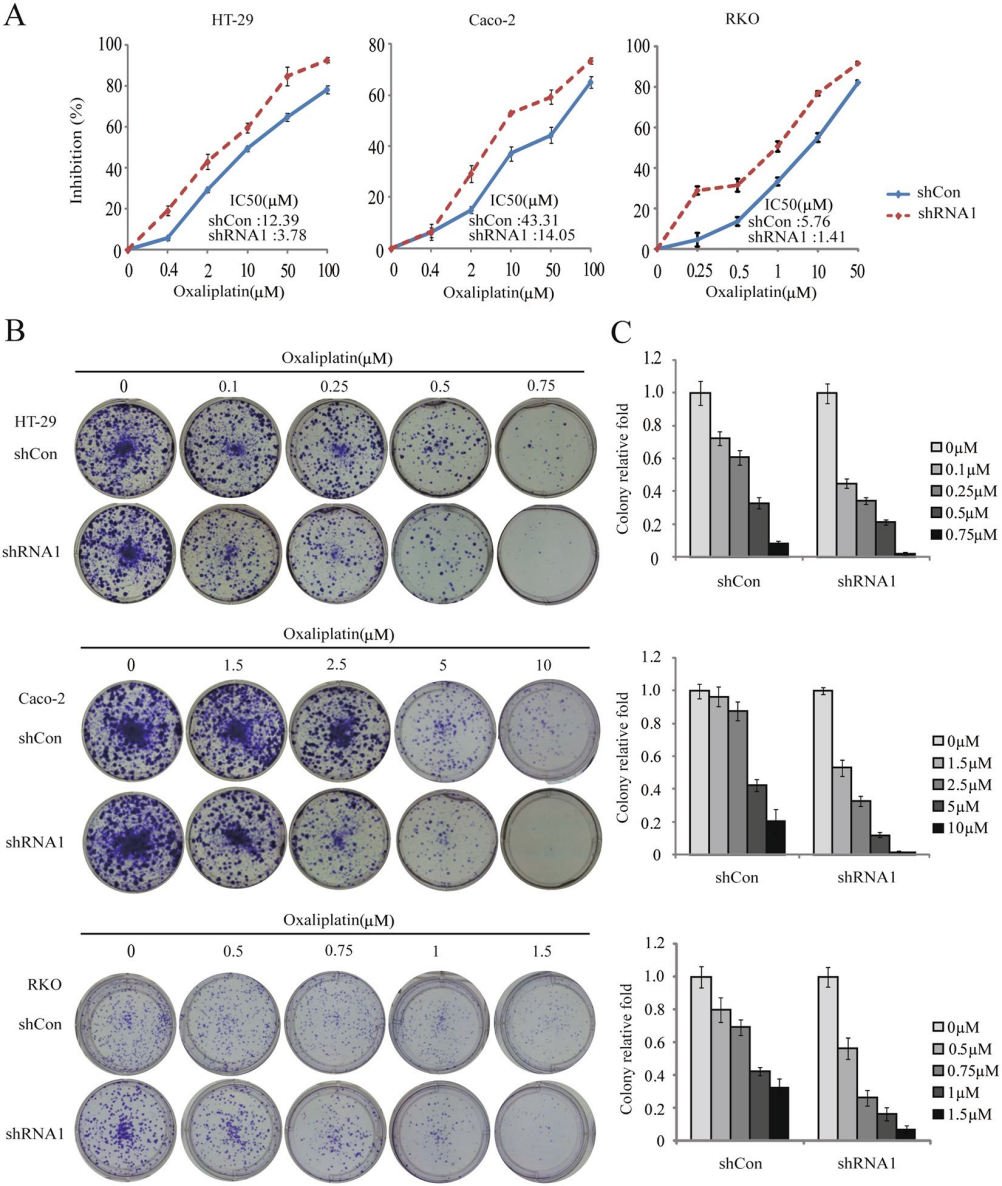
*WBSCR22* knockdown sensitized CRC cells to oxaliplatin. (**A**) Cell proliferation assays of the shCon and shRNA1 treated CRC cells upon oxaliplatin treatment. The IC_50_ value of each cell line was indicated. The results were the representative of at least three independent experiments and the results were expressed as the mean ± SD. (**B**) Colony formation of the shCon and shRNA1 treated cells in the presence of oxaliplatin. (**C**) Quantification of the colony formation of the shCon and shRNA1 treated cells. The results were expressed as the mean ± SD.

To investigate the effect of *WBSCR22* on oxaliplatin sensitivity, MTT and colony formation assays were performed. The cells pretreated with shCon and shRNA1 were incubated with oxaliplatin at various concentrations for 72 hours, and IC_50_ values were determined. The IC_50_ values for oxaliplatin in the shCon and shRNA1 treated cells were 12.39 ± 0.66 and 3.78 ± 0.14 μM for HT-29 cells; 43.31 ± 2.67 and 14.05 ± 1.09 μM for Caco-2 cells; 5.76 ± 0.20 and 1.41 ± 0.21 μM for RKO cells, respectively. In all three lines, the IC_50_ for the shRNA1 treated cells were significantly reduced compared to the shCon cells (3.28-fold, 3.08-fold and 4.09-fold decrease for HT-29, Caco-2 and RKO cells, respectively) (Fig. 3A), indicating that *WBSCR22* knockdown could sensitize CRC cells to oxaliplatin. Indeed, colony formation assays showed that the number of colonies upon oxaliplatin treatment for 48 hours was significantly decreased after *WBSCR22* knockdown (Fig. 3B,C). We also performed the assays in Caco-2 cells using an additional shRNA2. The efficiency of knockdown and the effects on oxaliplatin sensitivity were comparable to shRNA1 (Supplementary Fig. S2).

**Figure 4:**
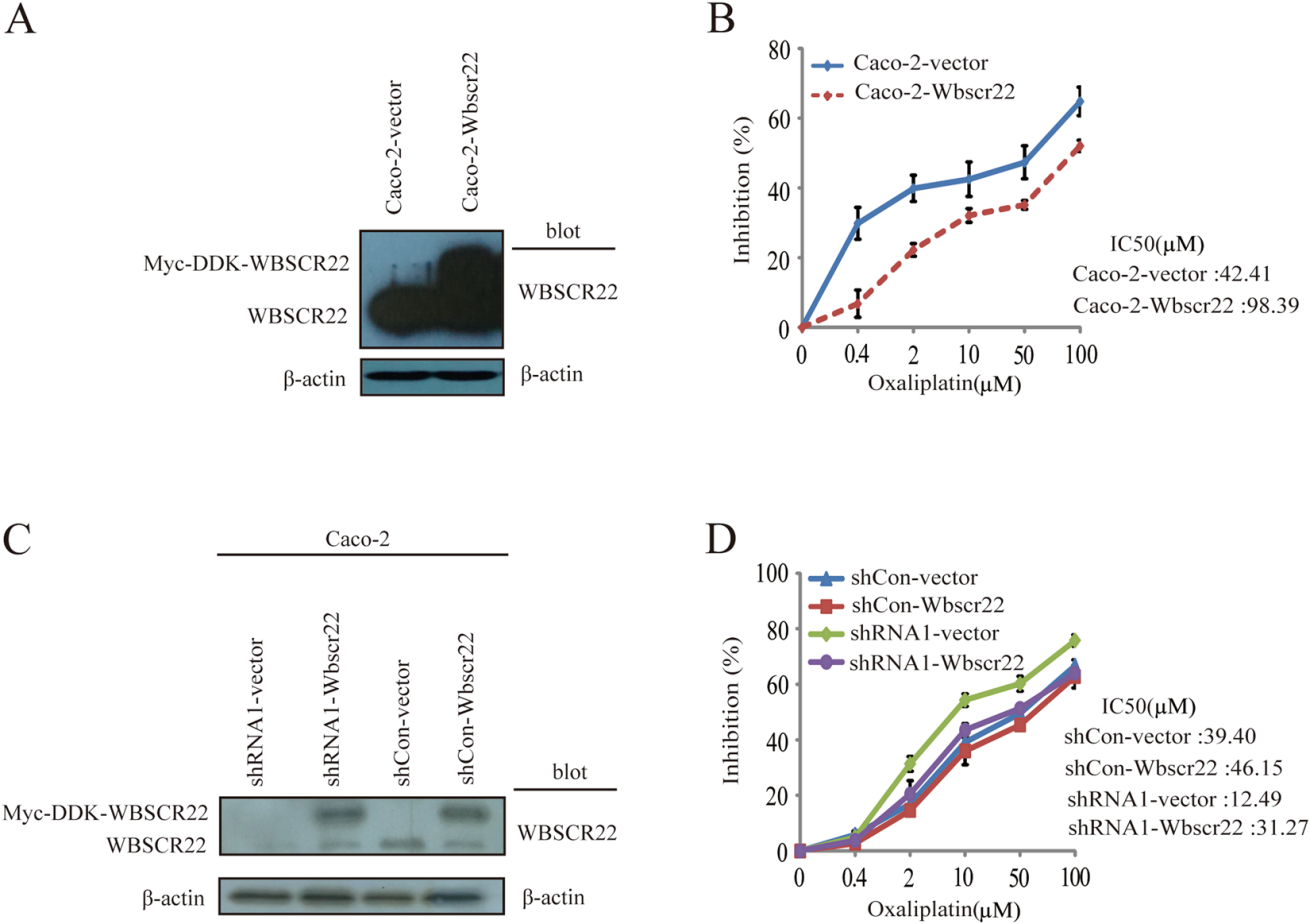
The expression of *WBSCR22* affected Caco-2 cell sensitivity to oxaliplatin. (**A**) Western blot analysis of WBSCR22 in Caco-2 cells transfected by Wbscr22 or vector constructs, respectively. (**B**) Proliferation study of the Caco-2-Wbscr22 and Caco-2-vector cells upon oxaliplatin treatment at the indicated concentration. The results were representative of three independent experiments and the results were expressed as the mean ± SD. (**C**) Western blot of WBSCR22 in the shCon or shRNA1 treated Caco-2 cells transfected by either Myc-DDK-WBSCR22 or vector constructs, respectively. (**D**) Cell proliferation of Caco-2 shRNA1-vector, shRNA1-Wbscr22, shCon-vector and shCon-Wbscr22 cells upon oxaliplatin treatment. The IC_50_ value of each cell line was indicated. The results were representative of three independent experiments and the results were expressed as the mean ± SD.

Importantly, overexpression of *WBSCR22* in Caco-2 cells (Caco-2-Wbscr22) led to a higher chemoresistance to oxaliplatin than the control (Caco-2-vetor). The IC_50_ values for oxaliplatin in Caco-2-Wbscr22 and Caco-2-vetor cells were 98.39 ± 8.79 and 42.41 ± 3.02 μM, respectively (Fig. 4A,B).

To further confirm that *WBSCR22* affects oxaliplatin sensitivity, we rescued the *WBSCR22* expression by transiently transfecting Myc-DDK-WBSCR22 construct or vector control into the Caco-2 shRNA1 cells (shRNA1-Wbscr22, shRNA1-vector) or shCon cells (shCon-Wbscr22, shCon-vector). Interestingly, the restoration of WBSCR22 protein decreased the sensitivity of shRNA1-Wbscr22 cell to oxaliplatin, indicating the elevated *WBSCR22* conferred the resistance of CRC cell to oxaliplatin (Fig. 4C,D).

### *WBSCR22* konckdown promoted the oxaliplatin-induced apoptosis in CRC cells

**Figure 5:**
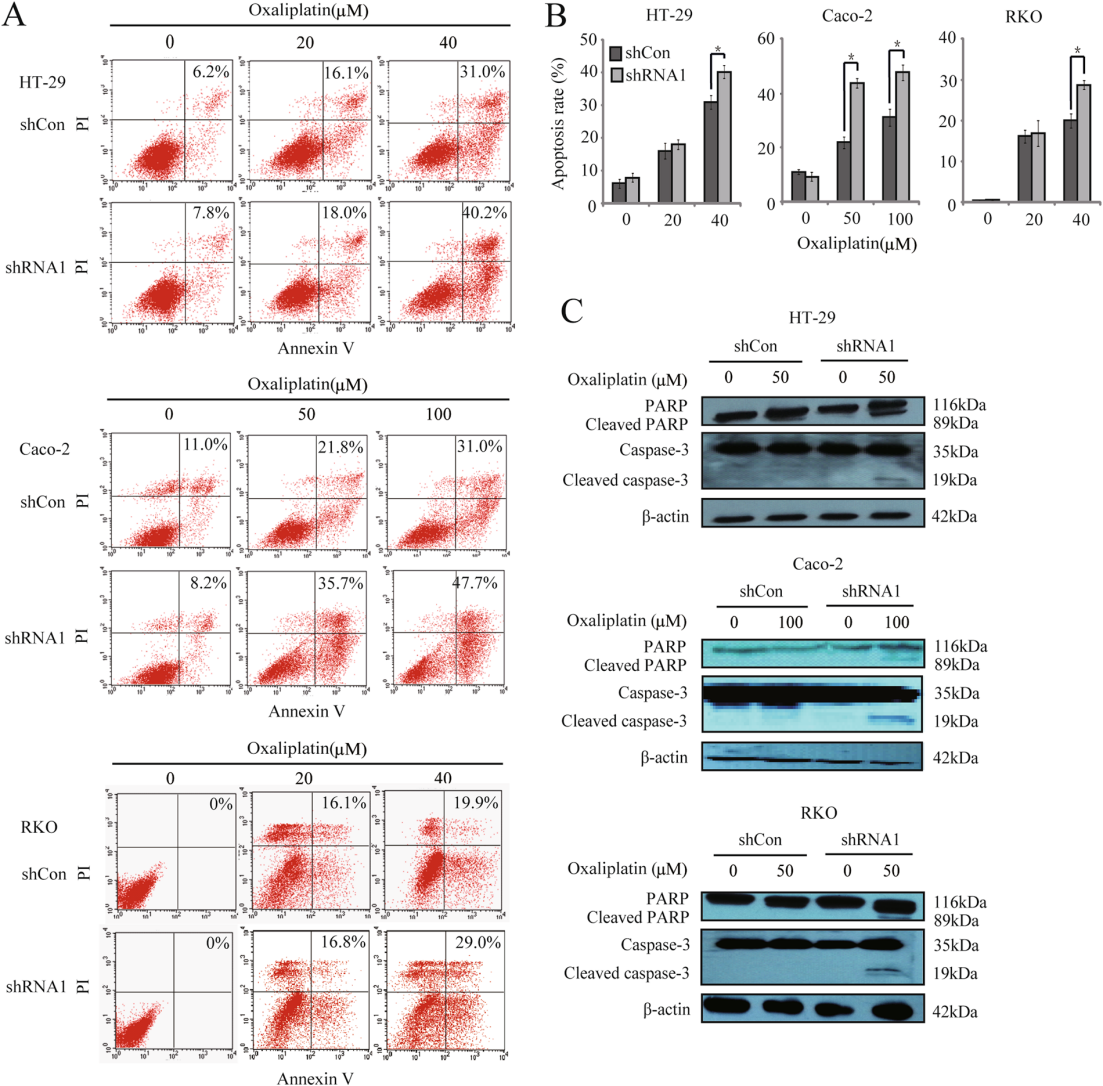
*WBSCR22* knockdown increased the oxaliplatin-induced apoptosis in CRC cells. (**A**,**B**) Cellular apoptosis rate was detected by annexinV-PI assay after oxaliplatin treatment for 48 hours. The rate of apoptosis was indicated. *p < 0.05 (shRNA1 v.s. shCon). The results were from three independent experiments and the data were expressed as the mean ± SD. (**C**) Western blot of cleaved PARP and cleaved caspase-3 in CRC cells after oxaliplatin treatment for 48 hours.

We further investigated whether *WBSCR22* knockdown affects the oxaliplatin-induced apoptosis. The shRNA1 treatd cells showed an increased rate of apoptosis upon oxaliplatin treatment at 48 hours (Fig. 5A,B). The apoptosis markers, both cleaved PARP and cleaved caspase-3 fragments induced by oxaliplatin, were detected in the shRNA1 treated cells but not in the shCon treated cells (Fig. 5C), indicating that the oxaliplatin-induced apoptosis was enhanced by knockdown of *WBSCR22* in CRC cells.

### *WBSCR22* knockdown increased the CRC sensitivity to oxaliplatin *in vivo*

**Figure 6:**
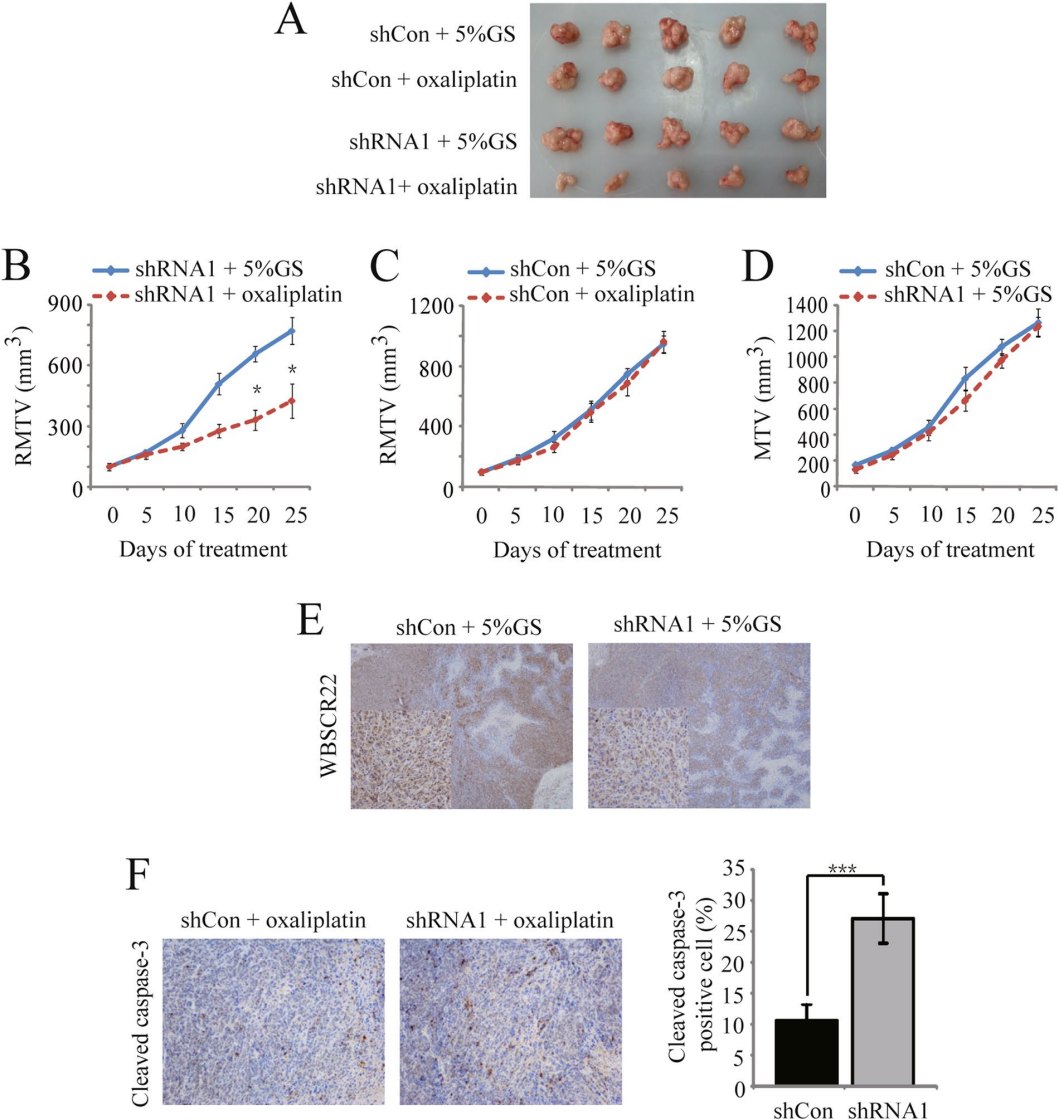
*WBSCR22* knockdown increased oxaliplatin sensitivity *in vivo*. The mice in the treatment groups were treated with oxaliplatin by intraperitoneal injection, on a schedule of two injections every week at 7.5 mg/kg per injection. The mice in the control groups were injected with solvent (aqueous glucose 5% solution [5% GS]). (**A**) Macroscopic images of xenografted tumors excised at day 25. (**B**) The relative mean tumor volume (RMTV) curve of the Caco-2 shRNA1 xenograft models. The data were expressed as the mean ± SE. *p < 0.05, (Caco-2 shRNA1 + oxaliplatin [treatment group] vs. Caco-2 shRNA1 + 5% GS [control group]). (**C**) The relative mean tumor volume (RMTV) curve of the Caco-2 shCon xenograft models. The results were expressed as the mean ± SE. (**D**) The mean tumor volume (MTV) curve of the Caco-2 shCon and shRNA1 xenograft models in the control groups. The data were expressed as the mean ± SE. (**E**) Immunohistochemical analysis of WBSCR22 protein in the excised tumor tissues from the shCon and shRNA1 control groups (×40, ×200). (**F**) Immunohistochemical analysis of cleaved caspase-3 in the excised tumor tissues from the shCon and shRNA1 treatment groups (×200). The data were expressed as the mean ± SD. ***p < 0.001 (shRNA1 + oxaliplatin [treatment group] vs. shCon + oxaliplatin [treatment group]).

To assess whether *WBSCR22* knockdown affects CRC sensitivity to oxaliplatin *in vivo*, we compared the tumor growth inhibitory activity of oxaliplatin in the Caco-2-shCon and -shRNA1 tumor xenograft models. All of the nude mice bearing the tumors survived during the treatment. As shown in Fig. 6A,B, the relative mean tumor volume (RMTV) was significantly smaller in the treatment group of Caco-2-shRNA1 model (481.50 ± 84.49 mm^3^) at day 25, as compared to the control group (772.70 ± 66.27 mm^3^; p < 0.05) with the inhibition rate (IR) of 45.08%. There were no differences in the Caco-2-shCon model between the treatment group (962.70 ± 73.28 mm^3^) and the control group (949.61 ± 56.50 mm^3^) (Fig. 6C). The results demonstrated *WBSCR22* knockdown could sensitize the Caco-2-shRNA1 xenograft tumor to oxaliplatin. There were no significant differences for the Caco-2-shCon and -shRNA1 xenograft mice in the control groups (p > 0.05) (Fig. 6D).

Immunohistochemical analysis of the xenografted tumor tissues at day 25 showed that the Caco-2-shRNA1 tumor had a lower level of WBSCR22 protein compared with the Caco-2-shCon tissues (Fig. 6E). The Caco-2-shRNA1 tissue in the treatment group had more cleaved caspase-3-positive cells than the control group (p < 0.001) (Fig. 6F), further confirming that *WBSCR22* knockdown increased the CRC cell sensitivity to oxaliplatin *in vivo*.

### *WBSCR22* knockdown increased the oxaliplatin-induced intracellular ROS generation in CRC cells

**Figure 7:**
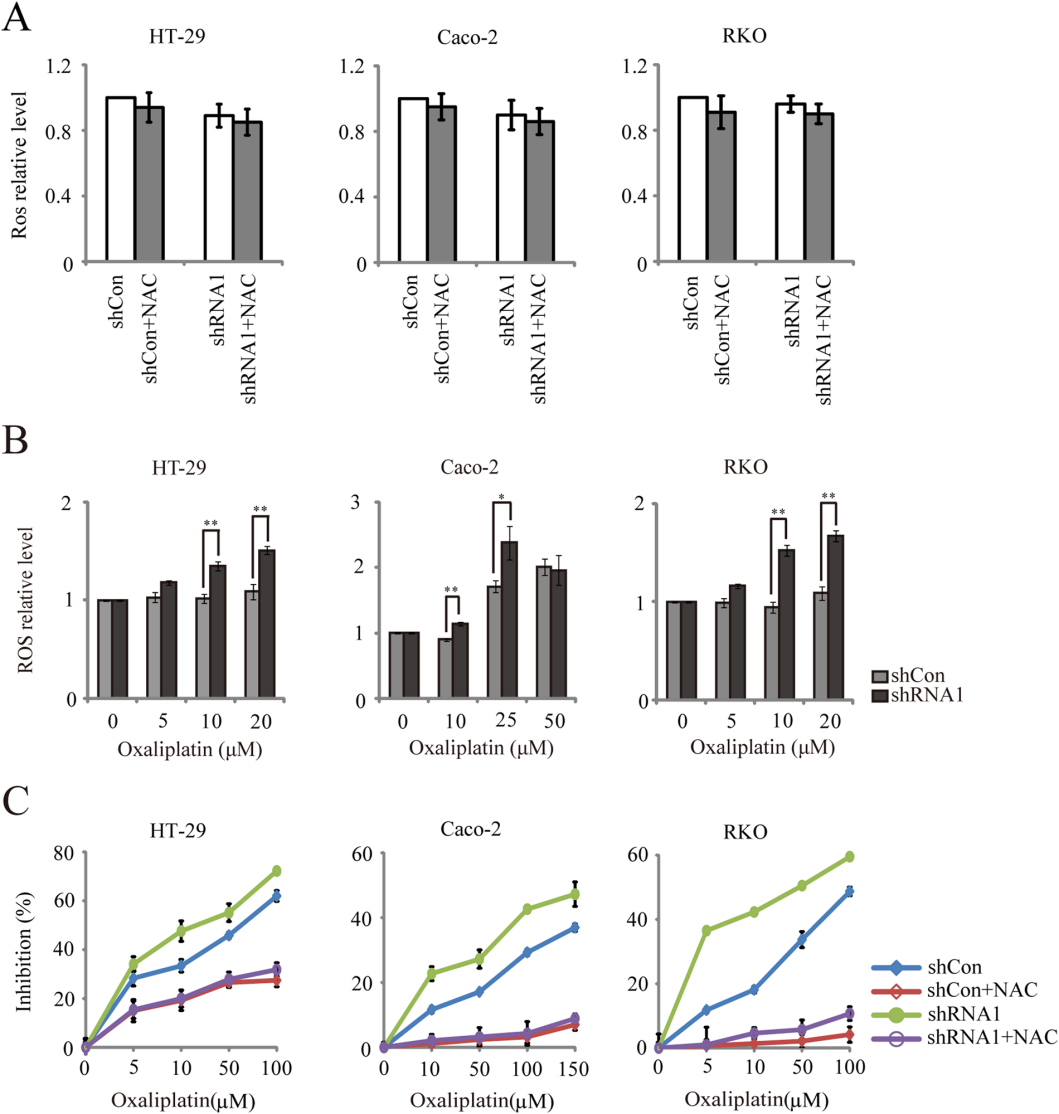
*WBSCR22* knockdown increased oxaliplatin-induced ROS generation in CRC cells. (**A**) Comparison of ROS levels in the CRC cells treated by shRNA1 and shCon. Cells were pretreated with 20 mM NAC or serum–free DMEM for 30 minutes. (**B**) HT-29, Caco-2 and RKO cells were treated with oxaliplatin at the indicated concentrations for 1 hour, and DCFH fluorescence was quantified immediately by a fluorescence microplate reader. *p < 0.05, **p < 0.01, (shRNA1 v.s. shCon). (**C**) Comparison of the oxaliplatin-induced cell proliferation in the shCon and shRNA1 treated CRC cells. Cells were pretreated with 5 mM NAC or serum-free DMEM for 1 hour, followed by oxaliplatin treatment for 24 hours. All results were representative of three independent experiments and the data were expressed as the mean ± SD.

ROS generation is known to be a critical event in the oxaliplatin-induced cell death. To investigate whether *WBSCR22* knockdown regulates intracellular ROS generation, we measured the ROS levels. The results showed no significant changes for the ROS levels in the shRNA1 treated cells as compared to the shCon treated cells. Treatment of these cells with nonspecific free radical scavenger N-Acetyl-cysteine (NAC) for 30 minutes did not significantly reduce the ROS production (Fig. 7A), suggesting that *WBSCR22* gene was not protective against the ROS generation in CRC cell lines.

We also quantified the levels of the oxaliplatin-induced intracellular ROS in CRC cells. As shown in Fig. 7B, there was a marked increase in the ROS level in the shRNA1 treated cells after 1 hour treatment with oxaliplatin, as compared to the shCon treated cells (HT-29 and RKO cells at 10 μM or 20 μM concentrations of oxaliplatin, Caco-2 cells at 10 μM or 25 μM), consistent with the results from the fluorescence image (Supplementary Fig. S3). Pretreatment with 5 mM NAC for 1 hour significantly protected both shCon- and shRNA1-treated cells against the toxicity of oxaliplatin (Fig. 7C). Our results demonstrated that *WBSCR22* knockdown increased the oxaliplatin-induced intracellular ROS generation, thus enhancing oxaliplatin sensitivity.

### *WBSCR22* knockdown increased the ROS-induced 8-oxoG accumulation in CRC cells

**Figure 8:**
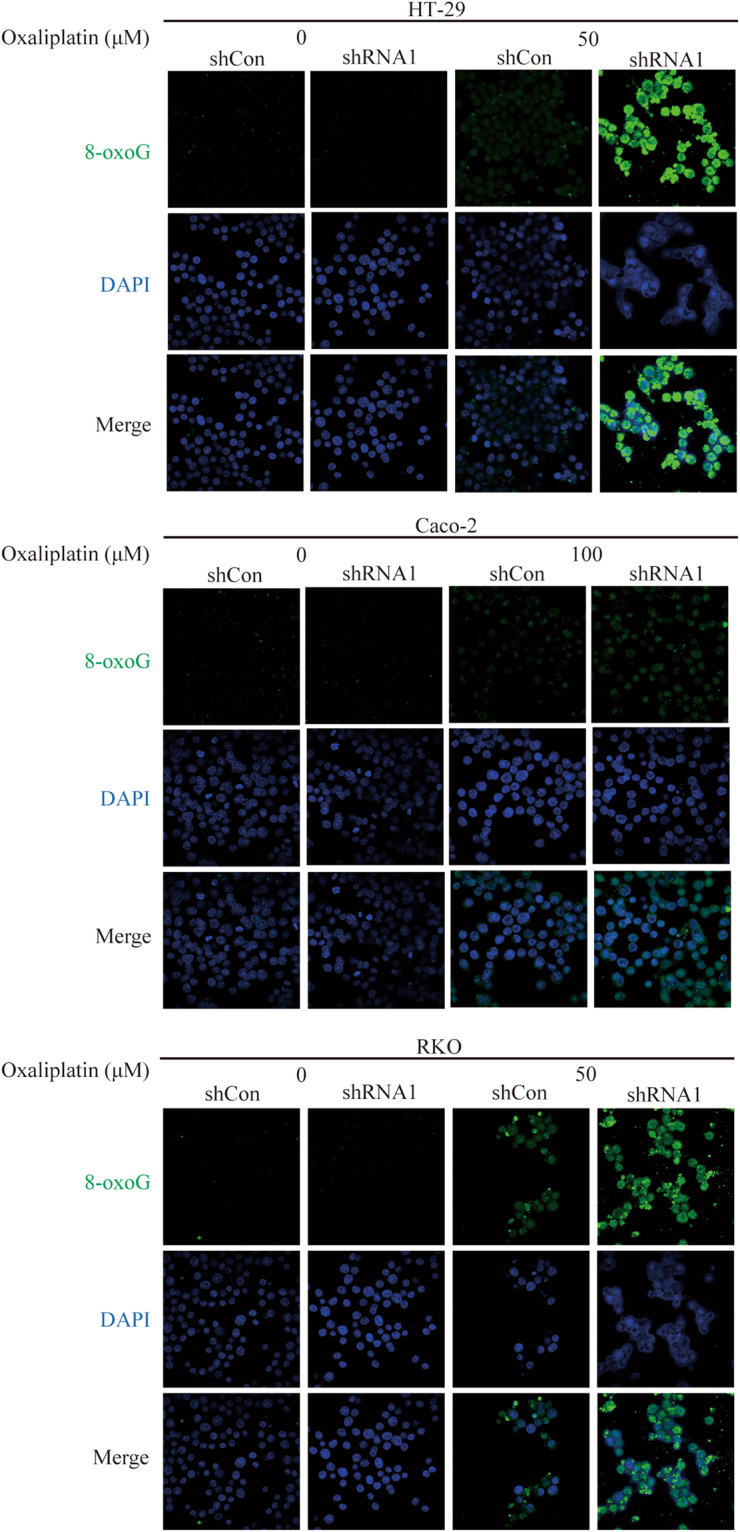
*WBSCR22* knockdown increased ROS-induced 8-oxoG accumulation in CRC cells. HT-29, Caco-2 and RKO cells were treated with oxaliplatin at the indicated concentrations for 24 hours. The intracellular 8-oxoG immunofluorescence (green) and cell nuclear DNA staining (blue) images were captured by a confocal laser scanning microscope (×400).

It was reported that oxaliplatin stimulates the intracellular ROS production, thus inducing the 8-oxoG oxidative lesion^19^. We therefore tested whether knockdown of *WBSCR22* could increase the accumulation of the ROS-induced 8-oxoG lesion by treating the cells with oxaliplatin. The intracellular 8-oxoG oxidative lesion was observed at the indicated concentrations for 24 hours by immunofluorescence staining (Fig. 8). Upon the oxaliplatin treatment, the shRNA1 treatd cells showed a brighter green fluorescence density (8-oxoG) compared to the the shCon treated cells, indicating that *WBSCR22* knockdown increased the oxaliplatin-induced 8-oxoG accumulation. The results were consistent with the elevated oxaliplatin-induced ROS level in the shRNA1 treated cells.

## Discussion

Oxaliplatin, a third-generation platinum antitumor drug, prevents DNA replication and transcription by forming both inter- and intra-strand cross-links in DNA, thus resulting in cell death. Oxaliplatin is non-cross-resistant with cisplatin and carboplatin^20^. The oxaliplatin chemoresistance is caused by multiple mechanisms, including overexpression and/or depletion of drug resistance associated proteins^12,21–23^, mutated genes^24^, altered platinum accumulation and DNA-Pt adduct formation^25–27^, microRNAs^28^, and defect in signal transduction pathway^29^. Therefore, how to overcome oxaliplatin resistance will be a key issue for more effective individualized therapeutic strategies.

Human *WBSCR22* gene is initially identified as a deleted gene in Williams-Beuren syndrome. Little is known about its biological function, particularly the impact on antitumor drug resistance. We have initially reported that *WBSCR22* knockdown attenuates the sensitivity of non-small cell lung cancer cell lines H460, harboring wild type *p53*, to 7-Ethyl-10-hydroxycamptothecin (SN-38; the active metabolite of camptothecin) and 5-fluorouracil (5-FU), but not in *p53*-null H1299 cells^30^.

In the present study, we found that CRC cells expressed high WBSCR22 protein, and the gene expression level of *WBSCR22* was correlated with the oxaliplatin sensitivity. Konckdown of *WBSCR22* significantly sensitized CRC cell to oxaliplatin *in vitro* and *in vivo* by increasing oxaliplatin-induced rate of apoptosis. Knockdown of *WBSCR22* significantly increased the production of oxaliplatin-induced ROS, thus simultaneously enhancing ROS-induced 8-oxoG oxidative lesion production. Pretreatment with NAC reversed the cytotoxicity of oxaliplatin in CRC parent cells as well as shRNA1 treated cells, indicating the oxaliplatin-induced ROS was a cytotoxic mechanism for oxaliplatin.

ROS are highly reactive oxygen free radicals or non-radical molecules that are generated by multiple mechanisms. ROS induce the 8-oxoG oxidative lesion production, as a pathogenic macromolecular lesion in the mechanism of platinum compound-induced cytotoxicity^31^. Oxaliplatin treatment destroys tumor cells in part through the induction of acute oxidative stress^32,33^. The production of ROS induces apoptosis and is involved in the oxaliplatin-induced cell death pathway^34,35^. Therefore, in our study the increased ROS and 8-oxoG level might sensitize human CRC cells to oxaliplatin. To our best knowledge, this study is the first demonstrating that *WBSCR22* is involved in oxaliplatin chemosensitivity.

In the future, the clinical samples should be collected to provide further evidence that *WBSCR22* is a potential prognostic biomarker and a promising therapeutic target for treating human CRC. These studies should focus on *WBSCR22* signal transduction pathways to further understand the molecular mechanism of oxaliplatain resistance, therefore designing therapeutic inhibitors to reverse its resistant effects.

In conclusion, our data showed that *WBSCR22* is involved in the CRC cell chemosensitivity to oxaliplatin, and CRC patients with lower *WBSCR22* expression might benefit more from receiving first-line oxaliplatin-based regimens than the patients with high *WBSCR22* expression. Our findings also strongly suggest that inhibition *WBSCR22* may be a potential therapeutic strategy for reducing colorectal tumor resistance to oxaliplatin treatment.

## Materials and Methods

### Analysis of TCGA data

Expression profile, clinical significance and prognostic value of *WBSCR22* were analyzed in the Caner Genome Atlas (TCGA)^36^ cohort. The data of gene expression and clinical information were obtained from TCGA data portal (http://tcga-data.nci.nih.gov/tcga/) and cBioPortal (http://www.cbioportal.org/public-portal/).

### Cell lines and reagents

The human colon cancer cell lines HT-29, Caco-2, RKO, HCT116 and Colo205 were purchased from cell bank (Shanghai Institute of Biological Sciences, Shanghai, China). Colo205, HT-29 and RKO grew as monolayers in Dulbecco’s modified Eagles medium (DMEM) containing 10% fetal calf serum (Gibco, Grand Island, NY, USA) in a 5% CO_2_, 95% at 37 °C. Caco-2 grew in DMEM containing 20% fetal calf serum (Gibco). HCT116 grew in McCoy’5 A (Sigma, Saint Louis, MO, USA) containing 10% fetal calf serum (Gibco). Oxaliplatin was purchased from Meilun Biological Co., Ltd. (Dalian, China). Oxaliplatin was dissolved at 2000 μM in 5% GS, as stocks and stored at −20 °C. Oxaliplatin stocks were diluted at a series of concentrations in serum-free DMEM immediately prior to use in the *in vitro* experiments. For the *in vivo* studies, oxaliplatin was dissolved at 0.625 g/l in 5% GS and 5% GS was used as the solvent control. N-Acetyl-cysteine (NAC) was purchased from Sigma. NAC was dissolved at 100 mM in serum-free DMEM, as stocks and stored at −20 °C.

### Plasmid construction and stable transfections

Plasmid construction and stable transfections were performed as described previously^30^. The shRNA sequence targeting human *WBSCR22* were 5′-GCCCTGTTACCTGCTGGAT-3′ (shRNA1) and 5′-GTCAGATGAAGGGCACTAT-3′ (shRNA2)^7^. HT-29, Caco-2 and RKO were stably transfected with vectors containing the shRNA sequence targeting human *WBSCR22* (shRNA1, shRNA2) or the negative control shRNA (shCon).

### Expression of *WBSCR22*

The cells were transiently transfected with *WBSCR22* plasmid (C-terminal Myc-DDK-Tagged) (OriGene, Rockville, MD, USA). The vector pCMV6-Entry (C-terminal Myc-DDK-Tagged) (OriGene) was used as the negative control. Transfections were performed using Lipofectamin™ 2000 (Gibco) according to the manufacturer’s instructions.

### RT-qPCR

Total RNA isolation, first-strand cDNA synthesis and qPCR were performed as described previously^30^. *β-actin* was used as a reference gene for qPCR analysis.

### Measurement of cell proliferation inhibition

The cell proliferation inhibition assay was carried out using MTT (Sigma). Cells were plated in 96-well plates at 1–5×10^3^ cells per well. At 24 hours later, oxaliplatin was added at the indicated concentrations for 24–72 hours. The absorbance was measured on a microplate reader at 570 nm. The IC_50_ values were calculated using GraphPad Prism 5.0 (GraphPad Software, Inc., La Jolla, CA, USA). The IC_50_ values were the means of at least three independent experiments.

### Colony formation assays

Cells were plated in 6-well plates at 5 × 10^2^ cells per well. After 24 hours, cells were treated with the indicated concentrations of oxaliplatin for 48 hours. Cells were then maintained in fresh medium for another 10 (HT-29 and Caco-2) or 5 (RKO) days. Colonies were stained with 0.1% crystal violet for 30 minutes and then counted.

### Western blotting

Cell lysis and western blot analysis were performed as described previously^30^. Antibodies used were as follows: anti-human WBSCR22 (GeneTex, Irvine, CA, USA), anti-actin, anti-PARP and anti-caspase-3 (Cell Signaling Technology, Inc., Danvers, MA, USA).

### Flow cytometry

The cell cycle distribution and apoptosis rate were analyzed by flow cytometry. For cell cycle distribution, 3 × 10^4^ cells were analyzed using a CytomicsTM FC 500 instrument (Beckman Coulter, Inc., Brea, CA, USA). ModiFit LT 3.1 trial cell cycle analysis software was used to determine the percentage of cells in each phase of the cell cycle. For apoptosis analysis, cells were harvested and washed with cold PBS after oxaliplatin treatment for 48 hours. The cells were then stained with annexinV-FITC and PI (Invitrogen Life Technologies, Grand Island, NY, USA), according to the manufacturer’s instructions.

### *In vivo* tumor growth inhibition assay

The animal study was approved by the Zhejiang Experimental Animal Center (Hangzhou, Zhejiang, China) under the project number: SCXK2008-0016, and the mice were maintained in accordance to the Institute Animal Ethical Committee guidelines approved by Zhejiang Academy of Medical Sciences (Hangzhou, Zhejiang, China). BALB/c nu/nu mice (male, 4–5 weeks) were housed in the laminar air-flow cabinets under pathogen-free conditions with a 14-hour light/10-hour dark schedule, and fed the autoclaved standard chow and water ad libitum. Both shCon treated and shRNA1 treated Caco-2 cells (2 × 10^6^ cells in 200 μl of serum-free DMEM) were subcutaneously injected into the right flank of mice. After the tumor volumes (TV) reached to 100 to 300 mm^3^ on day 8, Caco-2-shCon and -shRNA1 tumor xenograft mice were randomized into two groups (control group and treatment group) with five animals for each group. The mice in the treatment groups were treated with oxaliplatin by intraperitoneal (i.p) injection at 10 a.m. twice a week for 3 weeks, at a dose of 7.5 mg/kg per injection, and the mice in the control groups received solvent (5% GS). The TV was measured every 5 days during the treatment period (25 days). The TV was calculated using the formula: π/6 × (length × width^2^), where length = longest diameter and width = diameter perpendicular to length. The MTV, RMTV and IR were calculated. RMTV was calculated using the formula: MTV on day n (MTVn)/MTV on day 0 (MTV_0_). The IR was calculated using the formula: (1-RMTV in treatment group/RMTV in control group) ×100.

### Immunohistochemistry

Formalin fixed, paraffin embedded sections were deparaffinized by xylene and rehydrated. For antigen retrieval, the sections were placed in citrate buffer (pH 6.0) and heated in a microwave oven for 10 minutes. For immune peroxidase labeling, endogenous peroxidase was blocked by 0.3% H_2_O_2_ in methanol for 15 minutes at room temperature. The sections were then incubated overnight at 4 °C with primary antibody and washed with PBS containing 0.05% TrionX-100. Incubation with corresponding secondary antibody and the peroxidase-antiperoxidase complex were carried out for 30 minutes at room temperature. Immunoreactive site were visualized by 3, 3′-DAB. The slices were counterstained by hematoxylin. Antibodies were as follows: anti-human WBSCR22 (GeneTex) and anti-cleaved caspase-3 (Cell Signaling Technology, Inc.). Immunostaining were reviewed under a microscope at ×40 and ×200 magnification, and the images were captured using Leica DM2500 camera. The expressions of the cleaved caspase-3 were quantified by counting the number of immunopositive cells in three different fields per slide. All slides were reviewed independently by two experts.

### Measurement of the ROS production

Intracellular ROS levels were measured using a DCFH-DA assay. Briefly, 1 × 10^4^ cells/well (96-well plate) were allowed to adhere and treated with both DCFH-DA and oxaliplatin for 1 hour at 37 °C in 5% CO_2_. After treatment, the media was removed. The cells were washed with PBS, then added 100 μl/well PBS. ROS-dependent conversion of DCFH-DA to fluorescent product was quantified immediately by a fluorescence microplate reader (excitation 485 nm, emission 530 nm) and fluorescence images were captured by a fluorescence microscope.

### 8-oxoG damage assay

The cells grown in the glass bottom dishes were exposed to oxaliplatin for 24 hours then washed with PBS and fixed for 5 minutes at 4 °C with an ethanol: methanol (1:1; v/v) solution. Following three washes with PBS, cells were exposed to RNase A (DNase and Protease-free) solution (200 μg/ml; Thermo Scientific, Waltham, MA, USA) at 37 °C for 1 hour to digest RNAs. After a brief wash with PBS, fixed cells were treated with 5% bovine serum albumin in PBS for 1 hour at 20 °C and subsequently incubated overnight at 4 °C in primary antibody solution containing mouse anti-8-oxoG monoclonal IgM (1:2000; Abcam, Cambridge, UK), followed by a brief PBS washing and incubation with secondary antibody solution containing Alexa Fluor488-conjugated goat anti-mouse IgM (1:250, Sungenebiotech, Tianjin, China) for 2 hours. Cells nuclear DNA was stained using DAPI for 3 minutes. Images were captured using a confocal laser scanning microscope (Zeiss Lsm710; Carl Zeiss AG, Oberkochen, Germany). The excitation/emission wavelengths for Alexa Fluor488 and DAPI were 495 nm/519 nm and 340 nm/488 nm respectively.

### Statistical analysis

The associations between *WBSCR22* expression and clinicopathological parameters were analyzed using Chi-square tests. For survival analysis, OS was defined as the elapsed time between diagnosis and death or the last follow-up. Survival curves were estimated by the Kaplan-Meier method and compared using the log-rank test. To build a model for the prediction of survival, univariate and multivariate Cox proportional-hazards regression analyses were performed, in which clinical variables with p < 0.05 in univariate analysis were pooled into multivariate analysis. The data of the *in vitro* experiments were analyzed using the unpaired *t*-test and two-tailed *t*-test. The data of MTV in the xenograft models were analyzed using the repeated-measures analysis of variance. The data of RMTV at the end of treatment in the animal models were analyzed using the Mann-Whitney test. SPSS 22.0 software (SPSS Inc., Armonk, NY, USA) was used for all statistical analysis and p < 0.05 was considered statistically significant for all tests. Figures were plotted in SPSS 22.0 software (SPSS Inc.) or GraphPad Prism 5 (GraphPad Software, Inc.).

## Electronic supplementary material

Supplementary Information
