# Supplementary material for: WBSCR22 confers oxaliplatin resistance in human colorectal cancer
Source: Sci Rep. 2017 Nov 13;7:15443. doi: 10.1038/s41598-017-15749-z (PMC5684350; doi:10.1038/s41598-017-15749-z)
Supplement: Supplementary file 1 — Supplementary Information [file 41598_2017_15749_MOESM1_ESM.pdf]

WBSCR22 confers oxaliplatin resistance in human colorectal cancer

Dongmei Yan, Linglan Tu, Haining Yuan, Jianfei Fang, Liyan Cheng, Xiaoliang Zheng* and Xiaoju Wang*
The Center for Molecular Medicine, Zhejiang Academy of Medical Sciences, Hangzhou, 310013, Zhejiang, China
* wangxj@zjams.com.cn, zhengxl@zjams.com.cn

Supplementary information

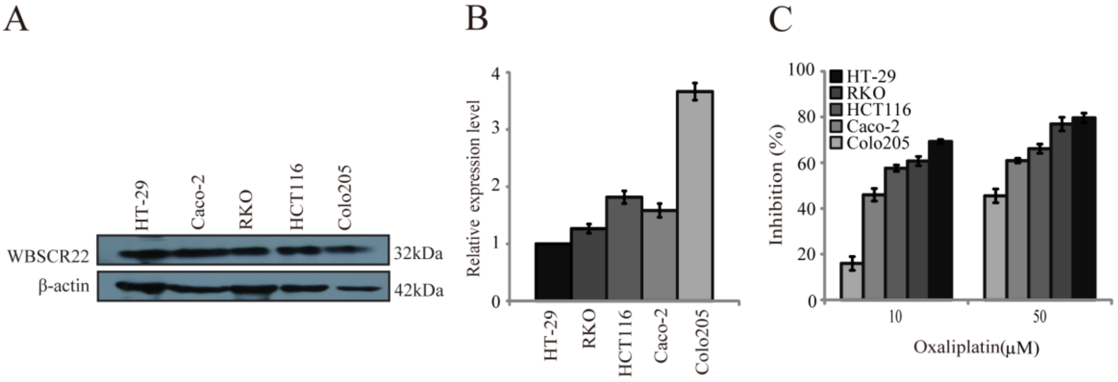

Supplementary figure S1. The expression of WBSCR22 at both protein and gene levels in human CRC cell lines with different oxaliplatin sensitivity. (**A**) Western blot of the endogenous WBSCR22 protein in CRC cells. (**B**) RT-qPCR analysis of *WBSCR22* gene in CRC cells. (**C**) Proliferation of various CRC cells in the presence of oxaliplatin. All results were representative of three independent experiments and the data were expressed as the mean ± SD.

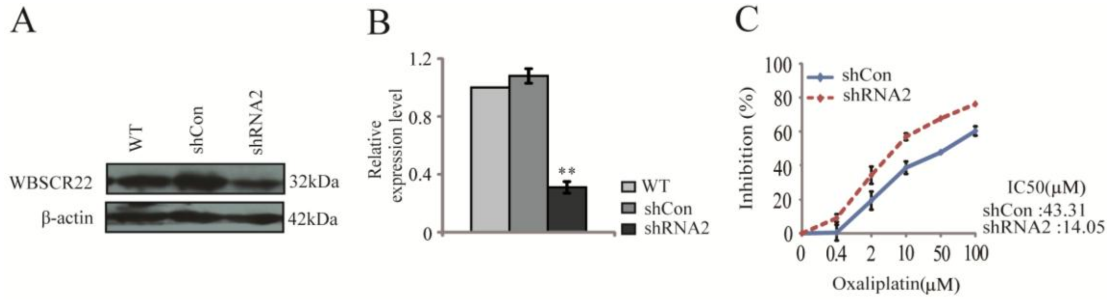

Supplementary figure S2. Knockdown of *WBSCR22* in Caco-2 cells by shRNA2. (**A**) Western blot analysis of WBSCR22 protein in WT, shCon, and shRNA2 treated Caco-2 cells. (**B**) RT-qPCR analysis of *WBSCR22* mRNA expression. **$p < 0.01$ (shRNA2 v.s. either WT or shCon). (**C**) Proliferation of the shCon and shRNA2 treated cells in the presence of oxaliplatin. All results were representative of three independent experiments and the data were expressed as the mean ± SD.

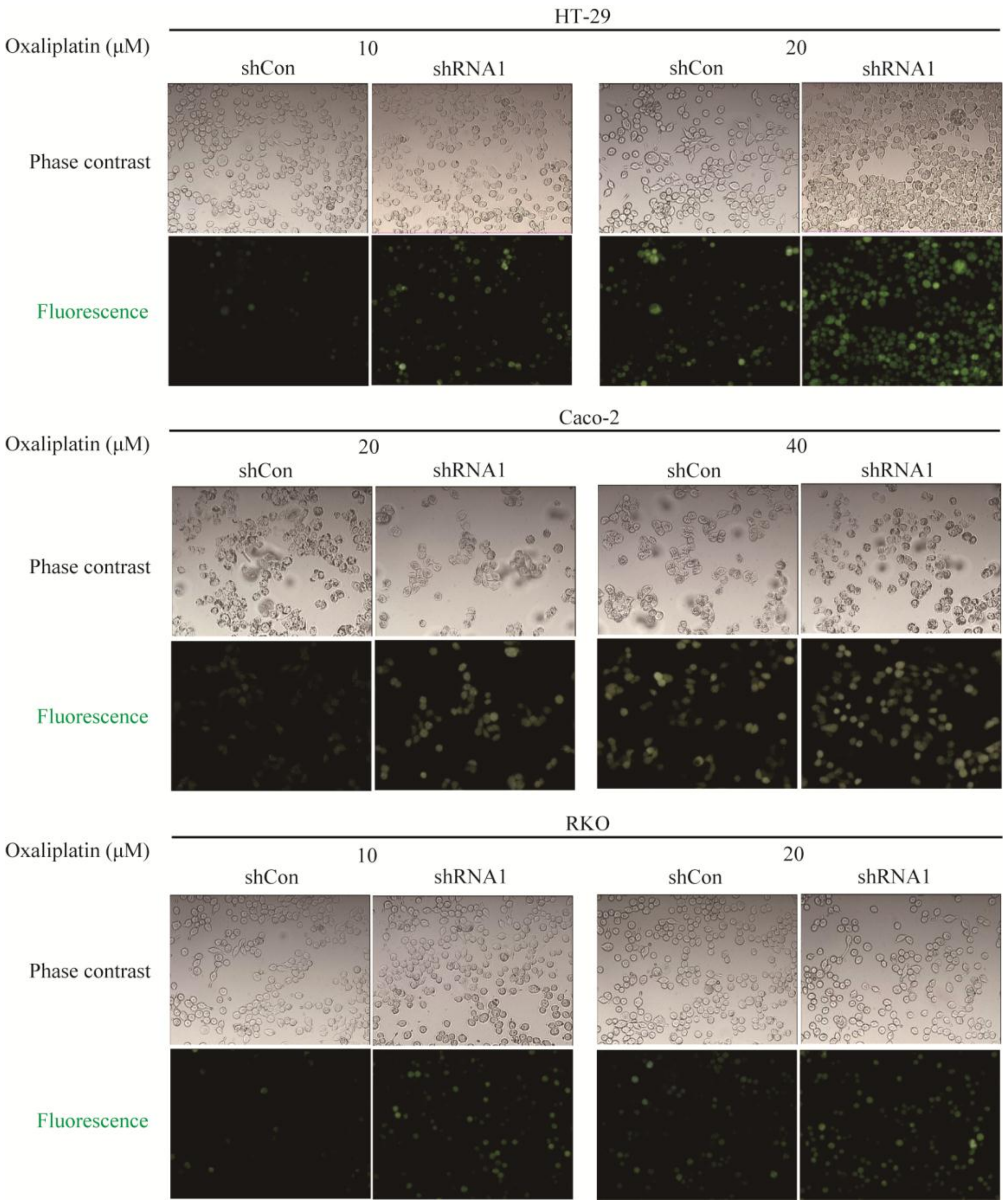

Supplementary figure S3. *WBSCR22* knockdown increased oxaliplatin-induced ROS generation in CRC cells. HT-29, Caco-2 and RKO cells were treated with oxaliplatin at the indicated concentrations for 1 hour. Intracellular DCFH fluorescence images were captured by a fluorescence microscope.

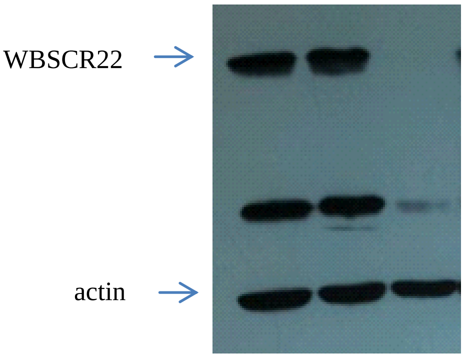

Fig. 2A HT-29

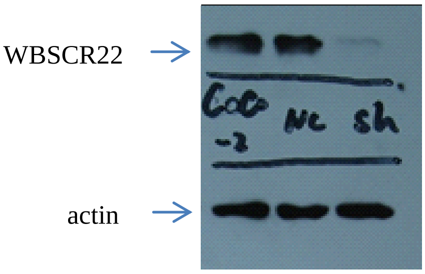

Fig. 2A Caco-2

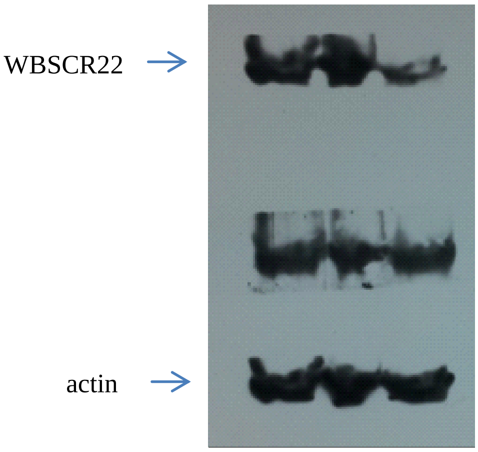

Fig. 2A RKO

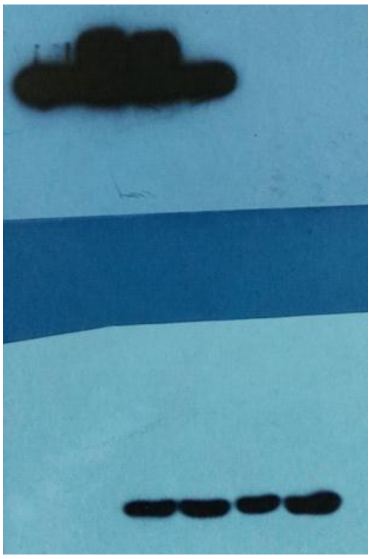

Fig. 4A

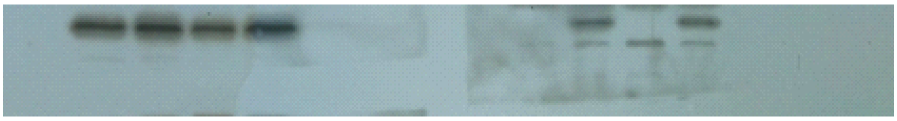

Fig. 4C

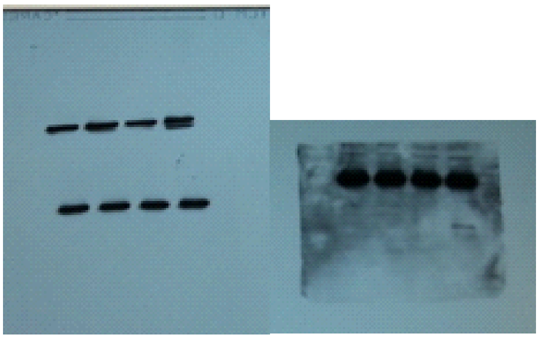

Fig. 5C HT-29

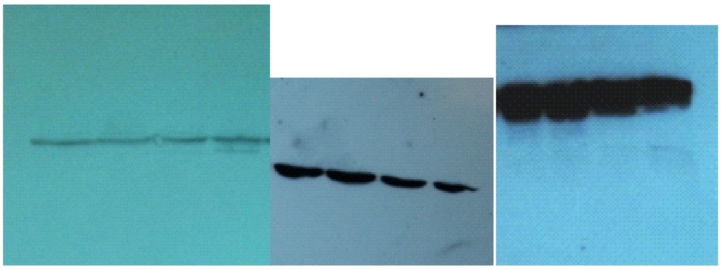

Fig. 5C Caco-2

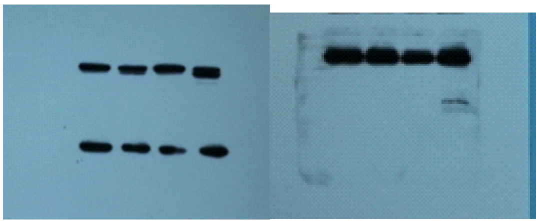

Fig. 5C RKO

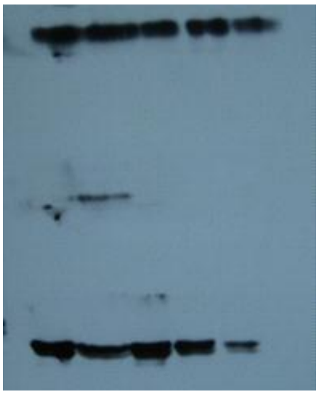

Fig. S1

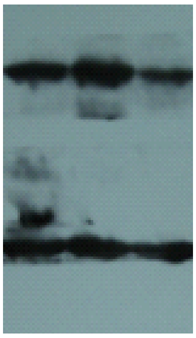

Fig. S2
